# A Hypothesis About the Origin of Biology

**DOI:** 10.1007/s11084-015-9426-5

**Published:** 2015-03-27

**Authors:** Neville J. Woolf

**Affiliations:** Steward Observatory, University of Arizona, Tucson, AZ 85750 USA

**Keywords:** Life, Autocatalysis, Membrane, NTP, Helix

## Abstract

It is proposed that processes characteristic of biology today, autocatalysis, selection of molecules for linkage by their electrical shape, and evolution by survival selection were also the processes that initiated biology. A reconnaissance is made of both paradoxes and potential questions. It is argued that the minimal requirement for initiating Darwinian evolution is not a molecule copying process, but a linkage copying process. Survival selection evolution does not require a heterocatalytic polymer and a separate replicase process until there is uncertainty where molecular additions will occur. It is argued that a linkage directing process will be found for a lipid membrane (though this needs to be verified) and may in the right environment result in initial evolution, including initiation of α-helices, the development of a single chirality and NTPs. The system has at this point become sufficiently complex that higher precision copying is needed. However it seems likely that this state is able to generate the first miniature ribozymes and their replicases, and so satisfies the prior requirement. With the proposed requirements, it is likely that the development of polymers was within membranes.

## Introduction

About 10 years ago, the late Lynn Margulis asked me “Where is the theory of the Origin of Biology?” She explained that she did not consider that a list of chemical reactions was an adequate explanation. I too consider that the development of biological processes involves issues of selection and linkage of molecules by their electrical shape, processes only otherwise found in crystal growth, though in both inorganic and organic chemistry. In this section we deal with the reason for writing the paper, and its goals. Below we shall discuss biological issues before continuing to paradoxes and further questions about life. Because life does not have an agreed definition, the transition from ingredients to a form that performs multiple survival-assisting tasks are here all considered to be part of the origin of biology, and this paper stops with an entry into a world where RNA is active.

The goal of this paper is to discuss mechanisms and potential mechanisms that may help resolve origin of life issues. It is now clear that the assumption by Oparin that life is an inevitable development is incorrect. Circumstances needed to match requirements for ingredients, energy supply, timescale and not being affected by bombardment by rocks, particles and X-rays, or needed components being on different sides of impenetrable layers of rock, water or ice. There had to be an extended development of the organism’s surface, nucleic acid control of bond making, and metabolic pathways, with the development sequence requiring interplay between these. The formation of life needed direction. With current understanding, that survival selection required a complex pre-development, and there seemed to be no adequately directive path to achieve this (discussed below in section “How Biological Processes Differ from Organic Chemistry”). The key proposals of this paper are that survival selection can operate with simpler conditions, and that survival selection drove the entire development process.

It is suggested here that there has been an imbalance between studying processes that can produce the chemicals of life outside an initial structure, a proto-organism, and processes inside it, with far more attention to processes outside. External processes form two groups. First there is the production of a huge variety of molecular forms. These are found in astronomical environments and Miller-Urey experiments. Their formation can be called “chemical production”. These molecules would have been available to proto-organisms and are not at issue. But also, nucleic acids and NTPs and their ingredient D-ribose, are at best rare. They require developments beyond chemical production. Processes for their production outside the proto-organism, and which can be studied in vitro have been studied as “chemical evolution”, external evolution. Processes that develop inside the initial structure are called “biological evolution”, internal evolution, and require studying in vivo, or rather, in membrano. This paper is at variance with some prior understanding of the words chemical and biological evolution, because here it is assumed they are potential alternatives for producing the same molecules and polymers. Internal processes have been less studied, partly because it has been believed that though survival guides development of simple organisms, that until genetic processes are available, developed benefits to proto-organisms cannot be continued to future generations, It will be proposed below, that benefits can be continued, while the development is simple, but as options for growth and change increases inside the proto-organism, survival benefit continuity does later require that a copying mechanism be developed. Spanning this transition phase is the main focus of this paper.

External processes could benefit from separate chemical environments which favored specific products. For example, the formose reaction might benefit from a warm lime pit. But then the separately produced products need next to be incorporated into the proto-organism by a process that Shapiro (1986) called “this little pool empties into that little pool” with an associated problem of dilution unless the products are encapsulated at the source. Further the “poisoning” of RNA growth processes with mixed chirality ribose, (Joyce 1987), implies that chiral issues needed to be resolved before RNA growth could self-explore. Yet organic chemical reactions do not readily produce single chirality products, whereas an enzyme-like process, selection for a molecule’s electrical shape, could more readily produce a single chirality ribose, provided that somehow the chiral enzyme was produced.

For internal processes there is the benefit that small molecules can easily penetrate the membrane, and the products of adding them will tend to be retained because of difficulty of the larger produced molecules crossing back out of the membrane. And once a single chirality is developed, a proto-organism could grow that way, and divide, so propagating the chirality.

Internal process studies need to show how to develop all the processes of life. This is contrary to the normal method of solution in both science and mathematics, where a problem is broken down into its components and they are solved by parts. But we do not know how Nature solved the development of life. Was it by parts, or was there a development in which membranes, amino acids and the components of nucleic acids assisted one another into forming polymers as is explored below?

This paper does not propose a totally internal process to add to chemical production. But that is an attractive option that deserves experimental study. At this conference Turk-MacLeod and Szathmáry reported that formaldehyde, H_2_CO, appears to perform a formose reaction inside a membrane. One might similarly explore whether HCN and/or formamide HCONH_2_, energized inside a membrane would produce nucleobases. These small organic molecules are among the most common. But in the interest of making a rapid exploration, it has been assumed here that nucleobases form outside the first membrane, even though they are of very low abundance in meteoritic matter. And it will be assumed that ribose initially formed by an external formose reaction, though its production as a single chiral form required an internal mechanism to replace it. Those mechanisms explored in this paper use membrane assisted synthesis to produce internal formation of NTPs, formation of a crude early cytoskeleton, internal formation of monochiral α-helices and through that, to monochiral developments that include initiation of the RNA World.

## How Biological Processes Differ from Organic Chemistry

Biological processes perform a set of reactions between organic compounds by using distinctive processes. Modern biochemical processes *grow* complex organisms, and so need additional steps beyond crystal growth. Reactions are commonly selected by *enzyme action*. Further, it is suggested here that terrestrial biological processes have a *special relationship to water*. A fifth aspect is that evolution itself has a necessary *sequence* in which there is first a separation of roles or functions, (explained below) and then optimization of the roles. These five aspects arise as follows:

1. Survival, as contrasted with slow degradation, is a situation in which there is growth by a process called “autocatalysis” (Troland 1916; Muller 1962) or “order from order” (Schrödinger 1944) or “dissipative structure” (Nicolis and Prigogine 1977). Autocatalysis is a process where an object or system uses the energy and material resources of the environment to grow itself, or something closely resembling itself. In this way it maintains itself through time, despite degradation processes. Autocatalytic processes occur both in living and non-living forms. Life, crystals, convection, forest fires, and hurricanes are all examples of autocatalytic processes. In such a process, both energy and necessary ingredients are made available by the environment, and the structure of the autocatalytic system organizes the growth, as e.g. by a crystal adding molecules so that they fit its electrical shape. Growth also permits parts to break off and grow independently, so it is the mother of reproduction. Thus parts of a crystal may break off, or a piece of a plant, and either can continue to grow.

Nicolis and Prigogine noted that autocatalysis results in dissipative structures, which are approximately steady states, resulting from environmental energy flow providing order that replaces the order lost to decay processes. All autocatalytic processes are dissipative, but dissipative structures also include non-autocatalytic processes, mechanical engines, refrigerators etc. Schrödinger’s term, “order from order”, seems to have been used only for the example of life, but it presents a view of life processes in which parasitic and symbiotic processes can be more easily understood. Order accumulates in living organisms, and so it is potentially available to drive other processes, especially other living processes.

Ordered accumulation of matter, without an energizing mechanism, is usually improbable because random processes and randomizing processes produce decay and dispersion. Despite that, the production of membranes and crystals require material but not additional energy. For crystals, the energy of attraction released from making molecular links is able to overcome thermal disordering processes, even though super-thermal processes such as convective motions may later break the crystals. For membranes the energy is instead, of electrical repulsion of lipids by water, with energy release as lipid structures grow. And membranes too may be broken. Both crystals and membranes add either individual appropriate molecules, or even smaller versions of themselves.

Other linkage reactions in life forms do require energizing, and require a cycle in which, at least for condensation reactions, polyphosphate is broken up and later phosphate is re-energized into a polyphosphate form. This energy flow was required for the development of life, and is why membrane-first models of the origin of life were classified by Shapiro (2007) as metabolism first processes. But a variety of processes of phosphate re-energizing, to complete the cycle, may have been used, and the focus here is not on those processes, but on the condensation reactions, and that which they energize. They are needed to make NTPs and RNA and an assortment of polypeptides.

2) Molecular evolution requires multivalent atoms so that a wide variety of compounds can form. Then the compounds can be incrementally varied, linked, or split, sometimes by substitution of small parts (functional groups), to improve survival probability. In particular, carbon’s nuclear charge and small inner electron core, allows it to link simultaneously to four different groups, and over a wide range of angles between them. So it gives rise to an extremely large range of compounds. Because of this, carbon is the preferred atom for making evolvable molecules (Pace 2001). Survival selection arises because population and/or individual growth (e.g. a banyan tree) limits resources, and therefore variant populations, distinguishable groupings and individuals are in competition with each other (Darwin 1859).
3) Because carbon bonding is so versatile, its molecules are correspondingly unselective. Carbon compounds need “help” in choosing their linkages. In biology, this help is provided by molecules that select by electrical shape, enzymes. Two molecules, which are the biochemical substrates to be linked, are held in their linked orientation, and commonly there is a “hinge” in the enzyme that links the substrates. The hinge thermal motion force differential motion of the substrates to be joined so that they move through the potential energy curve, and settle into the state of lowest energy, either bound or unbound. The weak binding of enzyme to substrate(s) is broken when bond formation occurs and the enzyme is freed to repeat its action.

In modern organisms most enzymes are large molecules, each specifically tailored by evolution to make one connection, but over and over again. Some small enzymes are promiscuous, performing a number of tasks. Yarus (2011) suggests that “early enzymes may have served a different function than today’s highly evolved, highly specific protein catalysts. Instead of furthering a pathway, or enforcing a highly specific fate for substrate, early catalysts may have facilitated many parallel reactions, and thereby allowed evolution to act on a variety of related products”.

Nonetheless it is necessary to explain how enzyme-like selective processes began. Yarus (2010) suggests that “The so-called ‘axiom of origin’ quantitates this notion; it presumes that the threshold for the RNA world can be approximately computed as the point at which sufficient random-sequence RNA exists to allow significant RNA activities to be selected”.

A measure of the difficulty of producing this threshold for the RNA world by random external reactions is seen by considering the tiny ribozyme GUGGC which aminoacylates GCCU from Phenylalanine-AMP as described by Yarus (2011). Even if we were to make a simplifying assumption that the only variables for making the ribozyme were selecting the form of the 5-carbon sugar, and the choice of the nucleobases for the nucleotides, there are 12 isomers of a pentose sugar, and within the isomers there are furanose, pyranose and linear forms. Also Joyce (2002) lists 11 potential nucleobases plus three substituted forms each of which would have multiple options. If we conservatively consider there to be only 12 variants of the sugar and only 12 variants of the nucleobases, the random assembly of a 5 nucleotide string would have over 61 billion variants, and only one would be GUGGC as we currently interpret those symbols.

It is the making of those selections, that seem to require enzyme-like paths to precede the production of this simplest known ribozyme. So, if D-ribose had already been selected, and only 4 nucleobases had been selected, the number of variants would be reduced to 1024, which would present no problem for further development. Even if we had a 25 % content of modified nucleobases, as in tRNA, the number of variants needed would only increase to 4315. The ratio of 61 billion and 4315 is about 2^24^. That is the selection of ingredients corresponds to making 24 sequential selections that each reduce the options by a factor 2, or somewhat fewer if some of the selections are by a factor 4, 8 or larger.

Yet Turk et al. (2011) state “*Before the evolution of modern proteins*, *initial peptide synthesis reactions, could not require protein enzymes. The discovery of RNA enzymes led to the RNA world hypothesis, which supposes that RNA was the sole biological macromolecule in early evolution. According to this hypothesis, early peptide synthesis was an RNA activity*”. But the transmission of a selective process to later generations is currently seen as requiring that not only must an enzyme perform the reaction, but there needs to be a process or molecule that copies the enzyme, a so-called “replicase”.

Since the numerical comparison above shows that it is extremely improbable that the RNA World could have been initiated without enzyme-like selection and assistance, and that initial simple survival development was without amino acid enzymes or RNA enzymes or replicases, it is instead suggested that it used a promiscuous enzyme like behavior of membranes, predicted and discussed in section “How Membrane Structures Performed the Roles of the First Enzymes, and What Did They Do?” to produce the first NTPs, structural proteins and RNA linkages. Such an ultra-simple prior replicating and copying system has been proposed many times, e.g. Joyce et al. (1987), most recently by Robertson and Joyce (2012) but it does not seem to have been considered that a membrane might serve this role.

4) Water was an available medium in which chemicals could interact at the start of life. Biological development seems to have made a virtue of necessity and now water serves two functions in life. The first is that water controls linkage processes, allowing specific reactions which are selected and energized. Other not-energized linkages are thermodynamically excluded. That is, without energizing, hydrolysis is favored over linkage.

Commonly, life uses chemical reactions that need energizing. Most of these are water producing reactions, “condensation reactions”, and because they produce water where water already is, these reactions require energy, usually from polyphosphate in the form of ATP. ATP uses up the water produced in the reaction, by the phosphate breaking itself in two, and so it assists condensation reactions. But then it needs re-energizing to make more reactions. Molecules remain unconnected until the combination of energizing and the necessary enzyme produced the condensation reaction.

The second role of water is in the formation and maintenance of membranes. In section “How Membrane Structures Performed the Roles of the First Enzymes, and What Did They Do?” it is argued that membranes in a water environment will direct which thermodynamically permitted linkages will occur and which not.

It is not yet clear whether a kind of life could develop in a different medium. The advantage of a polar medium is that it drives non-polar and semi-polar molecules together, regardless of their detailed composition, and so it assists evolution by keeping the products together. So life might well be impossible in the methane lakes of Titan because methane is not polar. Alternate polar media would seem more plausible

5) Evolution has a molecular sequence because specific functions arise by separation from a stem generalized activity. An example is how the simple autocatlytic process of membrane growth needed to be augmented, by membrane shape control and by nucleic acid copying to perform the more selective task of microbial reproduction.

Each time a new function developed it also became available for modification to assist some other survival assisting task, or as a start for another development. More complex molecular structures evolved from structures with fewer components and linkages. So there was a path of molecular development which we may trace, and so understand. Regardless of whether the early stage of a new development was neutral to survival or not, the continual selection for survival resulted in a web of reactions, in which some earlier developed molecular products, later became vital to survival.

## Paradoxes and Questions

There are three paradoxes that require resolution before understanding how life originated.

A) The first is the gene paradox, that if information copying is required for evolution, how did the first information copying evolve? (Section “The Gene Paradox”)
B) The second is the Muller paradox (1962), that the arrangement of chemical processes to produce autocatalysis is not trivial. So how is it that when a genetic autocatalytic process changes, the evolved process is also autocatalytic? (section “The Autocatalytic Paradox”)
C) Eigen (1971) argued that a complex autocatalytic process without precision copying would cease because of an error catastrophe. But one cannot expect an unevolved process to have precision. So how could the complexity of life begin? (section “The Absence of an Error Catastrophe Paradox”)

Following discussion of these, three questions are used to lead through processes and developments n needed for the “origin”, which is treated as the steps that lead into an RNA (plus structural protein) World:-

D) What was the range of pre-biological molecules available on early Earth. (section “The Variety of Pre-biological Molecules Available on Early Earth”)
E) How do membranes form? And how could or did membrane structures perform the roles of first enzymes? And what did they do? (section “How Membrane Structures Performed the Roles of the First Enzymes, and What Did They Do?”)
F) How did chiral biopolymers arise? (section “The First Linear Polymers”)

Handedness, chirality, enters the discussion of polymer production because it determines the direction of rotation of alpha helices and nucleic acids. The origin of chiral polymers was required to be at the beginning of the development of linear polymers. It would have led to the first processes for distinguishing, separating and using amino acids. The origin and start of development of linear polymers and their first use marks the transition between the origin of life and the development of genetic life. That will be the terminal development for this paper.

## The Gene Paradox

The gene paradox is, that if information copying by replication is required for survival selection evolution, how did information copying first evolve? The standard position, is that there was no biological evolution before genetic replication. It has been restated by Joyce (1987). “Two informational processes are fundamental to the operation of an evolving system. First the genetic information must be replicated in order to compensate for the inevitable loss of individual copies due to chemical degradation. Second the genetic information must be expressed as a behavioral phenotype so that its usefulness can be assessed by natural selection. In biology, both of these processes rely on the use of a polymerase enzyme.” Similarly Dobzhansky (1965) stated “In order to have natural selection, you have to have self-reproduction or self-replication, and at least two distinct self-replicating units or entities.” This need for two entities, is that one unit performs the copying, of both self and the other unit, and stays the same, and the other unit, which evolves, performs survival assisting tasks, so that it can be subject to natural selection, but it is copied so that the copier and evolver are both transmitted to future generations. It is this paradox, that complex units were needed before biological evolution could begin, that drove the concept of an alternative to biological evolution—chemical evolution, to produce the first evolvable genetic components.

I disagree with the premise and conclusion. My disagreement is not with the reality of random degradation, but that even at the beginning, genetic information must be generated to compensate for it. I deny that gene-like components are required for initial evolution. As stated, above, any autocatalytic process, genetic or not, can and will (competition permitting) compensate for random degradation. As seen from the Dobzhansky discussion above, it is the requirement for evolution as well as autocatalysis that creates a problem.

I stated at the conference that “Without genes, life had to start very simply. It had to start itself, be evolvable, and in the absence of genes and enzymes it had to be directive.” If one thinks only in terms of making copies of molecules, I believe the problem would be insoluble. But there is a stem activity (section “How Biological Processes Differ from Organic Chemistry”, aspect 5) which is the parent process to copying a molecule. It is internal modification by energy flow through the system. And a like process, in a like system, will tend to produce a like result. Thus the molecules produced by energy flow in the parent, will also be produced by energy flow in the descendant.

So *an approximate copy can be made, not only by copying the product, but alternately by copying its growth process*. This can be much simpler! And when growth is selective and directed, because of enzyme-like activity, inside the proto-organism, copying the process can lead to selective formation. An evolutionary process without a genetic core is a novel concept that requires discussion beyond that possible here.

As stated above in section “How Biological Processes Differ from Organic Chemistry”, aspect 3, it is because carbon is so versatile in its linkages that it requires highly selective processes be used to produce specific molecules. Evolution without enzyme-like processes, seems an improbable solution to the origin of biological evolution, because there are so many possibilities for organic chemical reactions.

Disagreement with the standard position has also been taken by Sowerby and Petersen (2002). “The origin of these self-replicating molecules also suffers from an informational paradox.” and “The odds are stacked against the random de novo appearance of exceptionally rare polymer sequences, and that begs for a deterministic rather than a stochastic origin. Such considerations inevitably force us to consider the possibility that life arose from simpler processes that did not rely on the primary involvement of linear, self-replicating molecules.”

If one were to imagine the simplest process for selective growth producing order, it would be that small molecules are added to one another and to the resultants, but each link is made in only one possible way. It would also be simpler if the process separated classes of molecules, amino acids were added to amino acids, and the components of NTPs were put together uniquely, and added to one another However it seems more likely that interconnecting processes were important too, because still today, amino acids begin their self-linking process by linking to an NTP. Also, it seems likely that α-helix amino acid chains would naturally develop as chiral, whereas there seems to be no such driver for NTPs. So there would need to be a reverse process too, where D-ribose was formed using amino acid and helix chirality to direct the formation.

Organic chemical processes commonly perform exothermic reactions, while biological ones are commonly endothermic before the use of phosphate, and use external energizing e.g. with ATP. While enzyme directed reactions allow survival selection to choose which links will be made, exothermic reactions will commonly produce an assortment of linkages. An example of this problem is in the formose reaction which is exothermic (Breslow 1959) where there are two sequential additions of formaldehyde to a diose sugar, which results in a tetrose which then breaks into two dioses, thus apparently reproducing the diose. In the process, one becomes two, becomes four etc. But unless there is some natural or human intervention, the formose reaction finds additional growth routes, and proceeds to make larger and larger polyols and on to form a chemical tar (Benner et al. 2010). It is possible to stabilize ribose with borate, aluminate or silicate, but the variety of potential products in such reactions, is mind boggling. It is therefore significant that, other than the manufacture of D-ribose, the reactions used by life to build the amino acid and nucleic acid polymers, are instead endothermic, in which dehydration occurs in an aqueous environment, and so the reactions are controlled by energy availability and its directed application. Benner et al. point to the possible resolution to the origin of ribose, that free carbohydrates were not biologically significant on Earth, rather they were linked to other molecules. An alternative is that those carbohydrates used were developed by an internal selection processes which added formaldehyde, molecule by molecule.

In the model/hypothesis discussed here, the gene paradox does not occur because sequential addition determined the early path of molecule development. Membranes formed spontaneously from meteoritic or cometary matter. Condensation reactions, driven by the availability of polyphosphate and re-energizing options for it, and the availability of glycerol (Pizzarello 2006), acted with polyphosphate in membrane molecules to make them become phospholipid. Further condensation reactions linked phosphate to ribose to nucleobases so making the first NTPs and used them to built crude cytoskeletons. Because these stronger membranes survived, they also selected whatever additional ingredients in the membranes that helped early NTPs (of both handedness) to form and produce the cytoskeletons.

Also these NTPs initiated processes of making α-helices, which solved a problem of the phospholipid membranes being relatively impermeable. However a-helices need to be monochiral to penetrate the membrane, and so the first chiral components developed. The preferred chirality of meteoritic amino acids led to production of membranes with only L- α-helices. It was then necessary to transfer this chirality to ribose, and so make monochiral NTPs. It seems necessary that the further D-ribose was manufactured in these protocells, somehow using the helices to select the way formaldehyde added to glycoaldehyde, giving D-glyceraldehyde etc. and produced D-ribose. (A directed formose reaction seems easier to control than a pentose phosphate pathway). In this way the NTPs became chiral, and the development of RNA became possible. Evidence of how the amino acids were selected for the helices still exists in the genetic code, and implicates uracil and adenine as needed (see section “The First Linear Polymers”). Then RNA linkage further suggests how G and C might be added.

At about this point the processes would have completed those selections, D-ribose and 4 nucleobases that were needed to bring the probability of production of tiny ribozymes into the realm of plausible numbers. The RNA world processes could begin. The proposal here is of necessity sketchy, and has many hidden issues, but it does face the need for a huge reduction in options before the RNA World could start.

The model here is similar to the model by Szostak et al. (2001), except that no replicase is needed initially, because the membrane is able to produce it by evolution, and also, needed ingredients could be gained from the environment.

Beyond the discussions of this paper were further developments that stabilized genes by making them with DNA, and improvement in making enzymes by making them from selected amino acids. The whole apparatus of modern reproduction and metabolism had to be developed, but the basis for it was available.

## The Autocatalytic Paradox

Muller (1962) has pointed out that the arrangement of chemical processes to produce autocatalysis is not trivial. So how is it that when a genetic autocatalytic process changes, the evolved process is also autocatalytic ?

“But the most remarkable feature of the situation is not this oft-noted autocatalytic action in itself—it is the fact that, when the structure of the gene becomes changed, through some “chance variation” the catalytic property of the gene may^1^ become correspondingly changed, in such a way as to leave it still auto-catalytic”. In other words the change in gene structure- accidental though it was-has resulted in a change of exactly appropriate nature in the catalytic reactions, so that the new reactions are adapted to produce more material like that in the new changed gene itself. It is this paradoxical phenomenon which is implied in the expression “change in the individual gene” or as it is often called, “mutation”.

The answer to Muller’s question has a number of components, but underlying them is this:

Simple inorganic autocatalytic systems, e.g. crystals or hurricanes and even membranes alone, have just a single growing unit (i.e. an NaCl crystal grows by addition of molecules, not the separate atoms). To build higher levels of structure, it is necessary for an autocatalytic unit to also perform heterocatalytic action. That is, it must catalyze additional reactions. Because the first reaction is autocatalytic, that would then link the heterocatalytic action to be part of the autocatalytic action. And then further heterocatalytic processes were also added. It is in this way that the reaction network of life was able to expand. That is the basis of the solution discussed here.

It is likely initially that autocatalytic processes produced membranes. We will see that, because membranes are pushed together objects, rather than autocatalytic because they pull together, they are able to be substantially modified and remain autocatalytic. And as noted by Budin and Szostak (2011), when the membrane acquires phospholipids in quantity, these are able to eject former membrane lipids such as monoacyl fatty acids. So survival improvement could occur.

Next is that the property of assembling other molecules into polymers, either by copying, or by repeating a standard pattern of linkage, is required to remain intact, even though that which is assembled changes. Changes to the polymer assembler would usually be expected to be lethal. This is why ribosomes have remained so stable over time that they can be used to create tree of life structures. However we shall find that the heterocatalytic property expected from membranes arises from the structure of the cytoplasm-membrane boundary, and so is likely independent of the exact form of the membrane.

Finally, evolution will favor producing molecules which not only perform a task, but which are also helpful to other needed tasks. That tends to change the mode of interaction of a subsidiary component from parasitic to symbiotic to essential. Originally membranes were autocatalytic and so formed. Because they constituted ordered structures, they were helpful as hosts to other processes, including ones that did not help survival. On that model, nucleic acid processes started as parasitic. Even today they do not directly assist the survival of their host membrane. Rather, they make components that assist the survival of their membrane. It was that making of survival-assisting components which evolved, and which made survive, those strong membranes which also supported nucleic acid processes. Though some parasitic processes continue to be supported, in general those processes we find today form a survival network. Those proto-organisms which did not develop a survival network of processes, and even those which were not efficient, failed to survive. The autocatalytic processes Muller found were just those processes which had passed the test of survival.

## The Absence of an Error Catastrophe Paradox

Eigen (1971) argued that in a multi-step autocatalytic process with n steps, that unless the precision of copying for each step were at least ~ (1–1/n), the process would decay. On the other hand it has been found that efficient evolution requires that copying precision be not too high, so as to have mutation test for optimum development. This is why observed systems sit close to the error catastrophe rate. However, in a natural system one would not expect precision copying, and so a first organism using an initial multi-step process for survival would be expected to fail. Polymer formation is such a multi-step process, and this is why it could not be a starting point.

However, the formation of a membrane is a 1-step autocatalytic process, so it is free from that problem. Amphiphilic molecules can add to a membrane one by one, or a complete micelle may be added. But the additions are independent and there is a complete unit with all needed properties after that one step.

All other processes that we will discuss, e.g. the development of the first α-helices, or the first miniature ribozyme required multiple steps to achieve a working unit. However, they arose as parasitic developments within a membrane, within a proto-organism that was already surviving. So completeness was not required for their survival.

If in any protocell population, the energy drain of a parasitic process was excessive, compared to its equivalent energetic support, that protocell population became extinct. But otherwise the development with increasing variations continued until the variation produced a new supportive process. Then the parasitism was transformed to symbiosis, and so became the basis for a further evolutionary development.

A model which starts with membrane autocatalysis is free from the Eigen error catastrophe. A model which begins with a muti-step production of a self-replicating polymer is not.

## The Variety of Pre-biological Molecules Available on Early Earth

Although the solar system started to form 4.57 billion years ago, the formation of planets was delayed, and the huge collision that produced a Moon and Earth occurred about 70 million years later, when Solar System volatiles were concentrated into icy and rock clumps. Since the heat of the Moon forming collision got rid of its initial atmosphere and oceans, they would have rid Earth of its initial volatiles too. The icy material falling in afterwards probably produced most of the volatiles to remake the atmosphere and oceans over a period of about ½ billion years, but already by 4.4 billion years ago, the Earth had water oceans and had a moderate temperature surface as shown by zircon studies (Mojzsis et al. 2010).

The volatile material that fell in, must have included the water carbon and nitrogen we now find here, though in unknown original forms. From comets and meteorites today e.g. Pizzarello and Shock (2010), we see that it also had assorted complex carbon containing molecules entering. To this mix, volcanoes on Earth were spewing out sulfur compounds and polyphosphate rich dust. When we compare these resources to the molecules needed for life, we see three different groupings. First, organic acids that could form membranes and amino acids were present in the infall. Secondly, phosphorus and sulfur were likely more readily available from volcanoes than from meteorites. Thirdly, nucleobases, nucleic acids and NTPs (nucleoside triphosphates) were not present or rare, likely because they are too reactive. Instead, materials from which they could be made, formaldehyde, formamide and hydrogen cyanide were present. We know this because we see these organic molecules in the interstellar clouds where new planetary systems are forming, and we also find them in comets and in some of the least heat-modified meteorites that land on Earth. There would have been a substantial aspect of the manufacture of nucleobases, sugars and NTPs in which proto-life processes produced the components. It is possible, even likely, that chemical reactions on Earth also produced organic molecules by Miller-Urey (Miller 1953) processes, but the need for them remains to be verified.

The forms from space were largely characterized by smaller molecules being more abundant than larger molecules. The stable forms from space included amino acids and soap-like organic acids. Although the majority of these are short chain and branched, there also appear to be longer chains not branched. Deamer (1985) discovered that the organic molecules in a meteorite, when in water could spontaneously form vesicles. Deamer and Pashley (1989) showed the variety of structures formed, and that long carbon chain organic acids were an essential part of this. Allen and Ponnamperuma (1967) suggest that the long chains might have arisen from surface interactions. Others have suggested Fischer Tropsch reactions. There is also the possibility that long carbon chains were produced by initially in space when acetylene molecules linking together as polyyenes including cyanopolyyenes (seen in space). Cyanopolyyenes, or polyenes (such molecules with carbon triple bonds broken and linked to hydrogen), in water might convert themselves to organic acids or amino acids by Strecker synthesis. The presence of all these ingredients suggest that a major metabolic development, such as developing the Krebs cycle was not needed until after life began. Prior chemical reactions were doing most of the work. The materials to make life were all present, but they needed organizing.

## How Membrane Structures Performed the Roles of the First Enzymes, and What Did They Do?

Since submission of this article, the author has become aware of prior discussions that touch on this topic, see Pohorille and Wilson (1995) and Böhler et al. (1996). There are two basic ways of driving growth. The growing matter may pull itself together, or it may be pushed together by their environment. Crystals energize their own formation because there is a binding energy. In contrast, Hurricanes are regions of low pressure which use external atmospheric pressure to push them together, until the moist air in the middle regions rises and rains out. Life seems to mainly use pushing processes, with most of the ingredients being pushed together, either by the watery environment or by enzymes. As a result, molecules that have one end polarizable sit with that end in the water, and the other end on a water surface. In water, two kinds of structure formed from part polarizable molecules are found. Micelles have the non-polar parts inside and make a kind of tiny bubble in the water. Vesicles make a double layer with water inside and water outside, and a thin oily layer between.

The structure of a membrane bilayer has been shown by Wiener and White (1992) to place the parts of the membrane molecules according to their polarizability. Their results an electric force field which stretches the molecules. There is a rapid force gradient at the membrane water boundary, and reduced force gradient away from it. The gradient places non-membrane molecules in positions where the force towards the water balances the force away from it. Individual functional groups are typically placed within a range of 0.4 nm in a boundary layer which is about 2 nm wide. It is this which results in selection for linkage as shewn next.

In a modern enzyme, molecules to be linked are held in all 6° of freedom, usually with some thermal flexing to exercise a potential bond. Contrast this with a membrane where the line of sight (Z axis) is into the plane of the membrane, and the membrane is spread in the X-Y plane. But a membrane holds a non-membrane molecule in 3° of freedom, with translation in the X-Y plane of the membrane occurring at fixed Z, and rotation about the Z axis also occurring. It is the fixing of the non-membrane molecule in the Z direction, and the inhibition of rotation around the X and Y axes that limits the possible linkages that can be made. All molecules where the Z depth of functional groups is similar will collide frequently, and so provided that energetics favors linkage, they will link. Those that do not match another in Z, remain unconnected to each other. This is part of the primary hypothesis of this paper, and it needs experimental verification.

In general, after a linkage between polar parts of two molecules has been made, the linked part is more hydrophobic than before and so migrates towards the membrane center. This leaves the new most-polar part of the combined molecule in a position for further linkage. Thus the joining of part polar molecules, as positioned by the membrane, can produce a linear polymer. The processes that need to have been aided by the membrane are all the early condensation reactions. These include phosphate being added to sugars and the products added to other molecules.

The origin of polyphosphate linked to sugars and their presence in protocells requires explanation. Polyphosphates are likely produced in volcanic dust (Yamagata et al. 1991), and conveying them to membranes through a water environment without their decay is not simple. Possibly, polyphosphates encapsulated themselves if they fell on an oil surface over water, which might develop an intermediate amphiphilic layer, which would curl up around the dust particles. Later disturbance of the water layer would precipitate these tiny membranes through the oil layer. Alternately, drying and rehydrating processes can move phosphate into a membrane.

Phosphate could link to the sugars if these passed through the encapsulating membrane. Glycerol is one of the more common sugar-like molecules in carbonaceous meteorites, and it would likely have been caught up in the process of making both cell and encapsulating membranes. Also it has been shown that ribose can pass through membranes (Sacerdote and Szostak 2005; Wei and Pohorille 2013). It is not clear whether the initial production of ribose was external or biological. If it were biological, the ribose to phosphate linkage would need to have used a cell membrane to bring the active sites together. One could expect formaldehyde to pass through a membrane, and so be available for an internal formose reaction.

The number of condensation reactions required for life to originate from modest sized molecules is huge. It is minimally one condensation reaction for every several carbon atoms. But the ratio of carbon to phosphorus atoms is about 100:1, and so the number of such reactions needed is far more than any plausible number of polyphosphate molecules originally present. Therefore the phosphate had to be re-energized. Today polyphosphates are largely re-energized by a complex enzyme system, ATP synthase. There must be an energy flow to re-energize phosphate. There are questions of whether this energy might re-energize with organic chemical processes, including use of thioesters (de Duve 1995) without enzymes, or even by photon absorption.

An alternate possibility discussed by Damer, Deamer and Norkus (2015) is that condensation reactions could be made by drying out the membrane. But then the ordering of which molecule links to which other, which is a result of orientation and position, produced by the water environment could be lost. For this reason it is assumed here that polyphosphate was both present with a re-energizing process, from the start. We will see that to make linkages polyphosphate first needed to operate with sugar-like molecules, and later, that increased directivity in linking amino acids required nucleobases too. Sugars were needed to bridge the hydrophobicity range from phosphate to other more hydrophobic molecules.

Because membranes made with monoacyl acids would have been weak, any process which converted them to stronger membranes would have had a survival benefit. And further conversion in any such survivor would have helped the survivors grow bigger and stronger. Some part of that process needed to be the growth of stronger membranes by adding in molecules from broken membranes and converting them too.

The simplest of the phosphate plus sugar added to another molecule, was the addition of polyphosphate to glycerol, abundant according to Pizzarello (2006), and then the linkage of this to a pair of monoacyl organic acids as discussed by Budin and Szostak (2011). This would have produced the first simple phospholipids. Phospholipids make much stronger membranes, and when they become the main membrane molecule they even eject single chain molecules from the membrane. However, the phospholipid membranes so created were far less permeable. So, because the transition to phospholipid membranes sealed protocells against transmission across the membrane, the development of a transmission path to permit materials for growth, and ejection of waste became essential. This was potentially the driver that selected the initial linkage of amino acids to make α-helices.

To link to amino acids, which are the second most abundant soluble molecular form in some carbonaceous meteorites, there is an additional task needed of bringing the OH from the sugar to the same level with respect to the membrane as the COOH of the amino acid, and with an appropriate orientation for linking. This seems to have been solved by making the sugar ribose, and by adding another molecule to it, a nucleobase which, by its hydrophobicity or hydrophilicity would accomplish both setting the level and setting the correct orientation.

It is likely that an early cytoskeleton was also made by the protocell. Membranes are relatively weak in holding together against shear forces. The strong bonds are perpendicular to the plane of the membrane. To make the membrane stronger, a structure is needed in which the bonds lie in the plane of the membrane. But first, the molecules to be linked must be pulled into the vicinity of the membrane. This requires that they be amphiphilic. Then there need to be multiple, at least two, functional groups in the plane of the membrane for each molecule so that lateral linkages can be produced. A number of molecular forms could satisfy this. Hydrophobic amino acids would suffice. Other options include hydroxy acids, and polycarboxylic acids. They would need to be bonded together by condensation reactions. Such a cytoskeleton would surely enter into the issue of how the first membranes divided when they became large. Today the cytoskeleton initiates the division by making a z-ring.

In this section we have seen that the presence of energized phosphate, sugars and some molecules that would adjust the height and orientation of the sugar bonds would result in stronger membranes. The linkages would have used these molecules, which were in form like NTPs. The second has not shown that these early NTP like molecules would form. That would preferably be examined by experiments.

## The First Linear Polymers

### Chirality

Joyce (1989) has pointed out that until a single handedness ribose had been developed, the origin of RNA would have been exceedingly difficult because of the way RNA of one handedness would have interacted with the other handedness. Now there are two ways that handedness enters into common biological polymers. In RNA the handedness comes from the use of D-ribose. In proteins it enters from the use of L-amino acids. If we recognize that natural ingredients provided both handedness, but biased to L-amino acids, then it would seem most reasonable that the amino acids selected handedness, and RNA followed suit.

The handedness of amino acids is important in making α-helices. The Wimley-White hydrophobicity scale (1996) shows that the peptide bond is itself polarizable, and would cause difficulty in insertion of hydrophobic amino acids through a membrane unless the bond is shielded, (Wimley and White 2000). In α-helices, the bond is shielded by virtue of the use of single handedness amino acids. The side group wraps around the helix and acts as a shield. Consequently only with a common handedness would α-helices self-insert.

These helices are structures that have a second interesting property. They can be made by linking random composition hydrophobic amino acids, which makes the amino acids amenable to being selected by membrane processes discussed here. However, because only linkages of common chirality amino acids will be able to insert themselves into and through membranes, a link between opposite chirality amino acids would seem to leave that bond open to destruction by hydrolysis and replacement by a same chirality amino acid. In this way it would seem that monochiral alpha helices which provide a path across the membrane may be self-developing. Not only would the path help the membrane grow, but by being a first purely chiral molecule for a membrane it might become a template for further chiral development.

The issues now become how amino acid chains grow, and whether this process itself would create monochiral helices. It is likely that the first linkage processes for an amino acid was a linkage to an NTP. The growth of an amino acid chain then requires these bonds to be broken. A possible process worth exploration is that there is an exchange reaction, which results in the initiation of both an amino acid chain and an initial RNA chain. This might be a process by which amino acid and NTP chirality became linked. Questions then arise about the linkages. Does an L amino acid attach itself as easily to L-RNA as to D-RNA? Do exchange reactions occur as easily between L-L and D-D pairs as mixed chirality? It could well be that the chain would keep on growing chiral. Experiments are needed.

Regardless of the processes, the probability of growing a monochiral chain would be strongly affected by the relative frequency of the two chiral varieties. Thus if the ratio of L to D were 0.55:0.45, and a 15 amino acid chain were required to bridge the membrane core, the probabilities of producing an L helix rather than a D helix would be 20 times higher. And if protocell development were to depend on the presence of such chains, then L-chain protocells would soon become the norm. However, unless the protocell itself started producing D-ribose, there would be a problem of NTPs and then RNA of both chiralities. As Joyce (1989) pointed out, the determination of chirality of necessity preceded the production of RNA polymers. Clearly some process terminated dual chirality fairly early in the development of polymers.

The questions above can best be tested by experiment, and they are the next group of required experiments to answer whether membrane evolutionary processes are the missing process prior to RNA origin as discussed in section “The Gene Paradox”. If the answer is in the affirmative, then it would seem likely that the origin of Biology was moderately simple as discussed here. If not, further processes must have entered.

### The Need for Nucleobases U and A

In an α-helix, the center which crosses the membrane is made of linked hydrophobic amino acids. At either end, hydrophilic amino acids link to the watery environments, and form N and C termini. These helices are the simplest structures that can be made with minimal selection of amino acids, but that selection, for hydrophobicity, is necessary. It appears that this selection can be achieved by the attachment of nucleobases to ribose-triphosphate. Of the five standard nucleobases in RNA and DNA, uracil is the most hydrophilic, and adenine is the most hydrophobic as inferred from bond polarizability, and so these bases will most pull or push on ribose-triphosphate when attached to it, either towards the membrane or away from it. The effect of this on linkage of hydrophobic versus hydrophilic amino acids needs to be checked, but the genetic code seems to show that the effect is used, as discussed below. Here we use the hydrophobicity scale of Engelman et al. (1986) because it is based on the partitioning of amino acids between membrane and cytoplasm.

In the RNA genetic code, U as middle letter codes for the most hydrophobic amino acids, phenylalanine, leucine, isoleucine, methionine and valine, and no others. On the Englemann scale these are all the most hydrophobic of the amino acids. Also, the START code is in this group.

A as middle letter, codes for the hydrophilic amino acids aspartic acid, lysine, glutamic acid, asparagine, glutamine, histidine, and tyrosine and no others. On the Englemann scale these are the most negative of the amino acids except that arginine is coded by G. The STOP code is also in this group.

Arginine is one of the amino acids not yet detected in meteorites, and it is also the most nitrogen rich of the biological amino acids. It would seem possible that this amino acid was never or rarely present in the material for the origin of life, and that it was later manufactured. These initial selections would have been retained during the further development of the code, and explain this aspect of the code today.

### Addition of G and C

In the discussion above, the nucleobases G and C have not had an obvious role. Guanine as noted by Eigen (1971), is a compound, that not only links to C but to other nuclobases. And in RNA, the normal Crick-Watson bonding of UA and GC tends to fail sometimes because U selects G.. Thus if (big *if*) there were a possibility of developing sequences of self-copying RNA with just U and A, then the later selection of G by U and the need for G to select a companion nucleobase to match the UA bond length would have brought in cytosine. There would have resulted in just the four standard RNA nucleobases.

This happy picture runs into the problem that the copying of short RNA chains is likely to have needed both G and C. And these two nucleobases could not have been used for initial hydrogen bonding of RNA chains before they were part of the chains. There is still the possibility that the earliest state of RNA was of UUU and AAA triplets and hydrogen bonded very poorly until first G, then C entered the mix.

The model of this paper, has then, that NTPs linked amino acids together, and it was the selection of amino acids to make trans-membrane proteins, α-helices that selected single chirality ribose for the NTPs. This interplay managed to produce small RNA groups that selected amino acids for trans-membrane helices. It was only later that they learned to copy themselves. The assumption that molecular addition was an adequately precise selector of development until RNA processes developed, is a hope, without either justifying or contrary evidence.

So far, molecules that performed some of these roles have been found, e.g. the tiny ribozyme. The smallest known replicase for it is currently larger, but perhaps a smaller version will be found. The key missing processes are how ATP and UTP both made an earlier selection of hydrophobic amino acids that linked to each other, and how the making of α-helices produced monochiral strings of amino acids as well as monochiral strings of nucleic acids.

The overall process proposed in this paper was a bootstrap one in which self-forming membranes started the selection process, and NTPs and RNA picked up the next stages. The development of the first genetic organism was a very complex sequence of events, and it is improbable that all the features suggested above will be correct. Experiments will suggest how they will need to be modified . But the basic suggestion of this paper, that genetics was an evolutionary development of existing Darwinian processes rather than the start of them, solves the paradox of the origin of the gene. The key advantage of the hypothesis that life was enzymatic from the very beginning is that it removes a number of improbable events and replaces them by directed growth of molecules and Darwinian selection.

## Conclusion

The goal of this paper has been to find a plausible route for the origin of biological organisms that avoided the gene paradox and Muller’s paradox. It is a reconnaissance, and raised many questions that need to be resolved by experiments. In this model, genes and genetic and reproductive apparatus are developments from a simpler stage in which role separation had not yet happened. The simplest initial state seems to be an autocatlytic membrane that is capable of evolution. Muller’s paradox does not apply to biological membranes, (section “Paradoxes and Questions”) and likewise the Eigen error catastrophe is avoided (section “The Gene Paradox”). It is proposed in section “How Biological Processes Differ from Organic Chemistry” that copying molecules was a development that began as a repeated process.

The second goal of the paper has been to understand how the selection of the ingredients of RNA came from an earlier stage of development. Some necessary features of the selection have been stated, and some arguments have been found that are plausible to the writer.

There are a number of places where it is suggested that experiments or modelling is needed. Some of these are for verification of the principal hypothesis, others are for points where suggestions were made to solve particular issues not in the direct line of the hypothesis.
